# Assessment of Mental, Emotional and Physical Stress through Analysis of Physiological Signals Using Smartphones

**DOI:** 10.3390/s151025607

**Published:** 2015-10-08

**Authors:** Inma Mohino-Herranz, Roberto Gil-Pita, Javier Ferreira, Manuel Rosa-Zurera, Fernando Seoane

**Affiliations:** 1 Department of Signal Theory and Communications, University of Alcala, Madrid 28871, Spain; E-Mails: roberto.gil@uah.es (R.G.-P.); manuel.rosa@uah.es (M.R.-Z.); 2 Faculty of Care Sciences, Working Life and Welfare, University of Boras, Boras 50190, Sweden; E-Mails: javier.ferreira@hb.se (J.F.); fernando.seoane@hb.se (F.S.); 3 School of Technology and Health, Royal Institute of Technology, Stockholm 14152, Sweden

**Keywords:** physiological measurements, smart textiles, smartphone, ECG, bioimpedance, stress detection, ergonomics

## Abstract

Determining the stress level of a subject in real time could be of special interest in certain professional activities to allow the monitoring of soldiers, pilots, emergency personnel and other professionals responsible for human lives. Assessment of current mental fitness for executing a task at hand might avoid unnecessary risks. To obtain this knowledge, two physiological measurements were recorded in this work using customized non-invasive wearable instrumentation that measures electrocardiogram (ECG) and thoracic electrical bioimpedance (TEB) signals. The relevant information from each measurement is extracted via evaluation of a reduced set of selected features. These features are primarily obtained from filtered and processed versions of the raw time measurements with calculations of certain statistical and descriptive parameters. Selection of the reduced set of features was performed using genetic algorithms, thus constraining the computational cost of the real-time implementation. Different classification approaches have been studied, but neural networks were chosen for this investigation because they represent a good tradeoff between the intelligence of the solution and computational complexity. Three different application scenarios were considered. In the first scenario, the proposed system is capable of distinguishing among different types of activity with a 21.2% probability error, for activities coded as neutral, emotional, mental and physical. In the second scenario, the proposed solution distinguishes among the three different emotional states of neutral, sadness and disgust, with a probability error of 4.8%. In the third scenario, the system is able to distinguish between low mental load and mental overload with a probability error of 32.3%. The computational cost was calculated, and the solution was implemented in commercially available Android-based smartphones. The results indicate that execution of such a monitoring solution is negligible compared to the nominal computational load of current smartphones.

## 1. Introduction

The literature includes numerous definitions for “stress”. This term was coined by Hans Selye [1,2], who defined it as “the non-specific response of the body to any demand for change”. In several studies, Selye observed that laboratory animals exposed to different noxious physical and emotional stimuli (e.g., extremes of heat or cold, perceptual frustration) all exhibited the same pathological changes or alterations, including enlargement of the adrenals and shrinkage of lymphoid tissue. In subsequent studies, Selye demonstrated that persistent stress could cause these animals to develop selected diseases similar to those observed in humans, such as stroke, heart attacks or kidney disease. This phenomenon is studied in detail by the American Institute of Stress [3].

To relate the stress and the physiological signals, it is necessary to examine the sympathetic nervous system, which is closely related to stress [4]. The sympathetic nervous system (SNS) is one of the three major components of the autonomicnervous system (ANS) in addition to the enteric and parasympathetic systems. The main focus of the SNS is to mobilize the body’s nervous system in what is known as the fight-or-flight response [5,6]. This response is a physiological reaction that occurs in response to a perceived harmful event, attack or threat to survival [7]. Moreover, this process is recognized as the first stage of a general adaptation syndrome that regulates stress response among vertebrates and other organisms. Therefore, the SNS is responsible for up- and down-regulating different homeostatic mechanisms. Certain reactions are provided by the SNS in such organs as the eyes, heart, lungs, blood vessels, sweat glands and digestive tract [8].

The knowledge derived from the real-time analysis of the stress level of a subject could be of special interest in the monitoring of many professional activities, such as those of soldiers, pilots, emergency personnel and other professionals responsible for human lives. Furthermore, assessment of professionals’ current mental fitness for executing the task at hand might avoid unnecessary risks. To obtain this knowledge, two physiological measurements were recorded in this work using customized non-invasive wearable instrumentation for electrocardiogram (ECG) and thoracic electrical bioimpedance (TEB) signals. In a previous work [9], the responses provided by the heart, sweat glands, temperature and the thoracic electrical bioimpedance signals were studied, and it was demonstrated that the best results were obtained for ECG and TEB measurements. For this reason, we use only these two physiological measurements in this study.

The designed customized wearable instrumentation consists of a vest, a recorder and a smartphone. The vest is comfortable, discreet and easy to wear and contains several electrodes that measure the ECG and TEB signals, which are recorded and transmitted in real time via Bluetooth by an ECGZ2 device to the smartphone, which is responsible for processing the data and estimating parameters related to monitoring of the SNS activity, such as the heart rate or the breathing rate.

Relevant information from both the ECG and TEB measurements is extracted by evaluation of a reduced set of selected features. These features are primarily obtained from filtered and processed versions of raw ECGZ2 measurements, which calculate selected statistical and descriptive parameters. The selection of the reduced set of features was performed using genetic algorithms, thus constraining the computational cost of the real-time implementation. Different classification approaches have been studied, but neural networks were chosen for this work because they represent a good tradeoff between the intelligence of the solution and computational complexity.

Three different analyses of application scenarios were performed to assess the performance of the proposed system. In the first scenario, the proposed system is capable of distinguishing among different types of activities that are neutral, emotional, mental and physical. In the second scenario, the proposed solution distinguishes among the three different emotional states of neutral, sadness and disgust. In the third scenario, the system is able to distinguish between low mental load and mental overload. The computational cost was calculated, and the solution was implemented in commercially available Android-based smartphones. The results indicate that the execution of such a monitoring solution is negligible compared to the nominal computational load of current smartphones.

The remainder of this paper is structured as follows. The hardware system is described in Section 2, and the software design is covered in Section 3. The generation and analysis of the experimental database are described in Section 4. Section 5 summarizes the results obtained by the proposed system with the generated database, and Section 6 provides conclusions for the paper.

## 2. Hardware System Overview

This section describes the different components of the designed system. The textrodes, the vest itself, the recorder unit and the smartphone.

**Figure 1:**
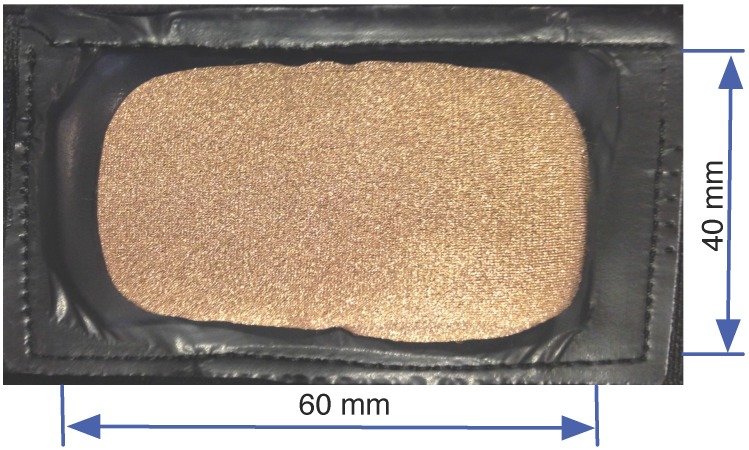
Textrode.

**Figure 2:**
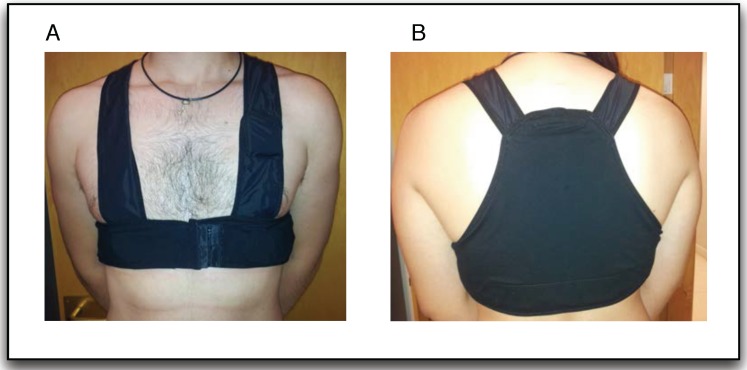
Vest: (**A**) front view; (**B**) back view.

**Figure 3:**
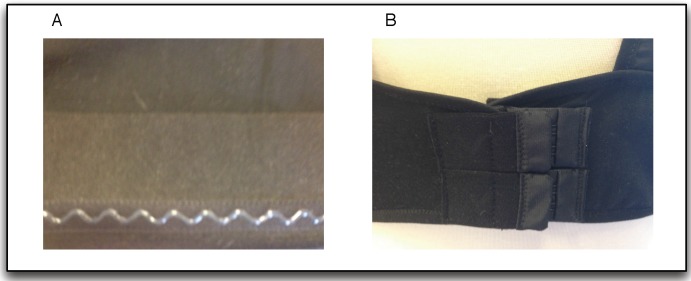
Vest: (**A**) anti-slip band; (**B**) adjustable fastener.

**Figure 4:**
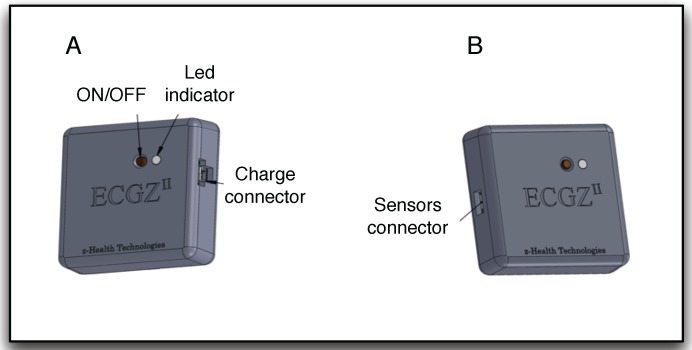
ECGZ2 device design: (**A**) left device view; (**B**) right device view.

- Textrodes: The textrodes are electrodes constructed with a textile structure that have an approximate surface of 60 × 40 mm and are used to measure both the ECG and TEB signal, using only four electrodes. These measurements are strongly related to the mental, emotional and physical state of the subject [10,11]. Figure 1 shows one of the textrodes that is included in the vest.
- Vest: The vest design is illustrated in Figure 2. Both comfort and usability were important characteristics in the design of the vest. To ensure a good grip, all contours of the vest are fitted with silicone bands. Furthermore, different sizes were created, and each vest includes adjustable options, as shown in Figure 3. Using these details, we provided a garment that is comfortable for both men and women.
- Recorder: To record the ECG and TEB measurements acquired by the textrodes, a device known as ECGZ2 was specifically designed to record and send data to the smartphone. The ECGZ2 allows sampling of each measurement with a different sampling frequency. For the ECG measurement, the sampling frequency is 250 Hz, and for the TEB, the sampling frequency is 100 Hz. This device is connected to the textrodes through wires included inside the vest. The device is placed in a pocket of the vest, and the design of this device is shown in Figure 4.
  In the previous version, the information was stored in Micro SD. However, in the new version, we send the information via Bluetooth to the smartphone, which is responsible for receiving and processing data and obtaining results in real time. Additional technical specifications can be found in [9].
- Smarthphone: The final component of the designed system is a smartphone, which is responsible for processing the information transmitted in real time by the ECGZ2 unit and estimating those parameters related to the monitoring of the SNS activity. In our design, the selected device was the “Samsung Galaxy Pocket” with an 832-MHz CPU, 512 MB RAM and a 1200-mAh battery. Section 3 describes the software included in the smartphone.

## 3. Software System Design

This section explains the details necessary for understanding the software system implemented in the smartphone and also describes the feature calculations for each measurement, the algorithms used to select the best reduced set of features, the performance of the classifiers with the selected set of features and each tool used to optimize the results.

**Figure 5:**
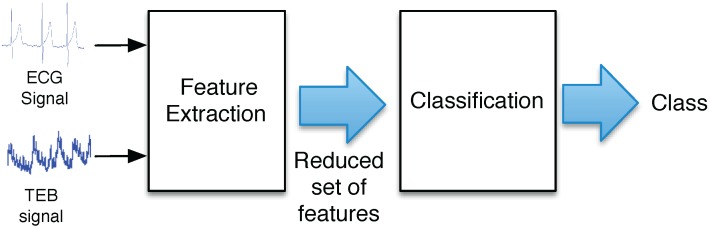
General scheme of the classifier.

The scheme used to obtain the complete software system is illustrated in Figure 5. The figure demonstrates that both the ECG and the TEB signals are the inputs of the system, and a reduced set of features is extracted from these signals, which is subsequently used as inputs to the classifier. In this work, the features are determined using a window of 60 s, and the classifier gives an output decision every 10 s.

It is important to note that to determine the set of features, both the performance of the classifier (in terms of error rate) and the computational complexity of the global system must be taken into account. For this purpose, a genetic algorithm tailored to this problem was selected. For simplicity, we consider the number of simple operations per second $N_{op}$ as an indicator of the computational complexity of the real-time implementation of a given set of features, because this value is proportional to the CPU load of the final implementation.

Thus, this section is structured as follows. First, the feature extraction process is described; the classifier specifications are explained; and a description of the algorithm is provided for the selection of the reduced set of features.

### 3.1. Feature Extraction

This section describes how the features are extracted from ECG and TEB measurements. This process refers to the block referred to as “feature extraction” in Figure 5. Special attention is focused on the description of the computational cost associated with the extraction of each feature. Therefore, all of the possible features are described, taking into account that the final real-time implementation extracts only a reduced number of features.

#### 3.1.1. Features for ECG Measurement

**Figure 6:**
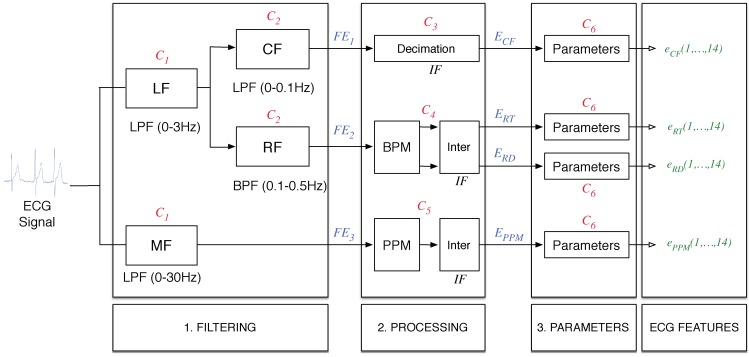
ECG-based feature extraction scheme.

Numerous types of features are commonly used to study ECG measurements, including heart rate variability (HRV) [12,13,14] or the power ratios of several frequency bands [15,16,17]. In this work, we use signals obtained and processed in three steps. Figure 6 shows the process used to calculate the ECG-based features in which three different steps can be distinguished: filtering, processing and calculating the parameters and baseline.

- Step 1: Filtering. The filtering step, as its name indicates, is a step in which the raw ECG measurement is filtered into different frequency bands. First, the signal is low-pass filtered with a cutoff frequency of 0.5 Hz (Low Frequency (LF) block) and then low-pass filtered with a cutoff frequency of 30 Hz (Medium Frequency (MF) block). In both cases, the filter order is $N_{1}=100$. The first filter is an anti-aliasing filter, which allows the use of interpolated finite impulse response (IFIR) filters [1]. Thus, the output of this anti-aliasing filter is applied to two different IFIR filters with a stretch factor of SF=25, both with order $N_{2}=1150$.
  The first IFIR (Continuous Frequency (CF) block) is a low-pass filter with a cutoff frequency of 0.1 Hz, and from this block, we obtain the first filtered signal ${\mathrm{FE}}_{1}$Ṫhe second block (Respiration Frequency (RF) block) is a band-pass filter with cutoff frequencies of 0.1 Hz and 0.5 Hz, from which the filtered signal ${\mathrm{FE}}_{2}$ is obtained. Finally, the last obtained filtered signal is ${\mathrm{FE}}_{3}$.
  Each filter in this step has a different computational cost due to the specific structure and the filter order. This computational cost is measured in the number of simple operations per second and affects the global number of operations in the features of the final system $N_{op}$. In this case, we use a filter with an order of $N_{1}=100$ for the LF and MF blocks. Thus, if the sampling frequency for the ECG measurement $F_{\mathrm{ECG}}$ is 250 Hz, then the number of simple operations per second is $C_{1}=N_{1}*F_{\mathrm{ECG}}=25,000$ operations per second. For the IFIR filters, the number of operations per second is denoted as $C_{2}$, and it is different due to the filter structure, because this filter is an IFIR filter, as shown in Equation (1):
  (1) $$C_{2}=\frac{N_{2}\cdot F_{\mathrm{ECG}}}{\mathrm{SF}}$$
  Thus, we obtain $C_{2}=11,500$ operations per second.
- Step 2: Processing. In this step, we used different algorithms for each signal obtained in Step 1. The signal ${\mathrm{FE}}_{1}$ is decimated to an intermediate frequency IF=50 Hz, using $C_{3}=3750$ operations per second. We selected this value because it is sufficient for extracting all information from the signal and allows standardization of the blocks in the next step.
  The signal known as ${\mathrm{FE}}_{2}$ is processed to obtain information from the breath. For this purpose, we use the block denominated beats per minute (BPM). This block considers the signal to be sine shaped, and it periodically determines the minimum and the maximum values of the signal, thus allowing estimation of the number of breaths per minute and the amplitude of these breaths. These values are used by the next block, denominated as “inter”, that interpolates the signals by generating two Intermediate Frequency (IF)signals using piecewise constant interpolation to the last known value. The first signal known as $E_{\mathrm{RT}}$ represents the breaths per minute interpolated to the IF, and the second signal known as $E_{\mathrm{RD}}$ represents the amplitude for each breath interpolated to the IF. This process takes $C_{4}=9050$ operations per second.
  The last signal provided by Step 2 is known as $E_{\mathrm{PPM}}$ and is obtained through evaluation of the pulsations per minute (PPM). The PPM block uses a simple algorithm based on thresholding of the five-sample differentiation of the signal ${\mathrm{FE}}_{3}$, thus estimating the position of the pulses using $C_{5}=8800$ operations per second.
  For the computational complexity of the blocks of this step, the number of simple operations required was evaluated for each block of the step.
- Step 3: Parameters. This step is necessary for obtaining the parameters that contain relevant information for monitoring the SNS. For this purpose, we calculated the 13 different parameters, listed in Table 1 with the corresponding required number of instructions per second $C_{6}$. It is important to note that in the case of the trimmed mean, the median and percentiles data must be sorted, which consumes nearly all of the operations required for the cases ($C_{6}=27,580$). Therefore, in the case in which several parameters of this type are evaluated for the same signal, the data must be sorted only once, thus reducing the number of operations.
  Furthermore, with the aim of characterizing the baseline of each signal, the last parameter denominated as baseline was calculated, totaling 14 parameters per signal. This baseline represents the long-term mean value of the signal and is calculated using a first order low-pass IIR (Infinite Impulse Response) filter. Because we use four signals ($E_{\mathrm{CF}}$, $E_{\mathrm{RT}}$, $E_{\mathrm{RD}}$ and $E_{\mathrm{PPM}}$) and 14 possible parameters, the number of available features for ECG measurement is 56.

**Table 1:** List of parameters and the number of operations per second required.

Parameter	$C_{6}$ (Operations Per Second)
Mean	300
Standard deviation	1201
Trimmed mean of 25%	27,805
Median	27,580
Skewness	2101
Kurtosis	2710
Maximum	300
Minimum	300
Percentile 25%	27,580
Percentile 75%	27,580
Geometric mean	3901
Harmonic mean	3301
Mean absolute deviation	1200
Baseline	550

#### 3.1.2. Features for TEB Measurement

**Figure 7:**
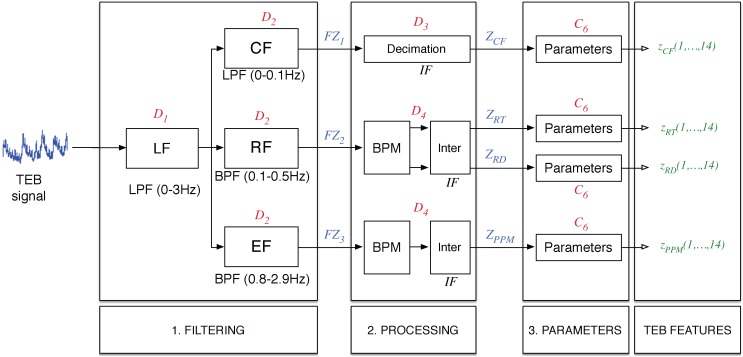
Thoracic electrical bioimpedance (TEB)-based feature extraction scheme.

For this second measurement, the procedure used to obtain the features is quite similar to the one used in the case of the ECG measurement, producing a total of 56 TEB-based features. Figure 7 shows the TEB features scheme, that again contains three different steps with a structure similar to that described for ECG.

For the sake of simplicity, only two significant differences in the blocks of the ECG are detailed. These differences are:

- The filters in the first step are different due to the use of a different sampling rates of $F_{\mathrm{TEB}}=100$ Hz. All signals from this step are determined using IFIR filters because the estimation of the PPM from the TEM is performed in a different manner.
- Because the shape of the pulse-related component in the TEB is completely different from the typical shape of the ECG signal, the algorithm for determining the PPM uses the BMP block, with parameters adapted to the properties of the signal.

This computational cost is different from the one obtained for the ECG-based features, because the sampling frequency is different, but equations similar to those described above can be used: $D_{1}=N_{1}\cdot F_{\mathrm{TEB}}$ and $D_{2}=N_{2}\cdot F_{\mathrm{TEB}}/\mathrm{SF}$, where now SF=10, $N_{1}=100$ and $N_{2}=400$. Thus, in the case of TEB, $D_{1}=10,000$, $D_{2}=4000$, $D_{3}=900$ and $D_{4}=2116$.

### 3.2. Classification

**Figure 8:**
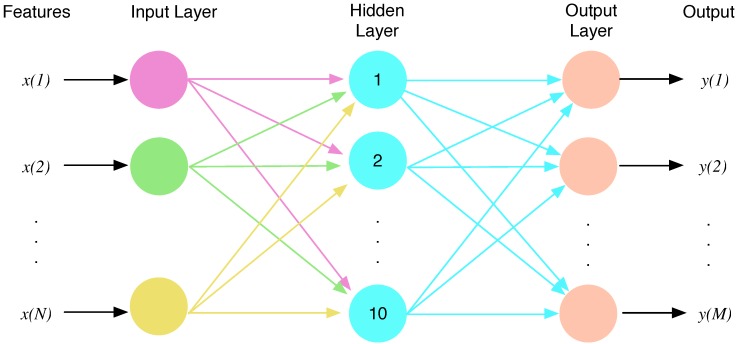
Multilayer perceptron scheme.

The classification process corresponds to the second block of Figure 5. The classifier block uses the input vector composed of the selected features in order to estimate the associated class, and several types of classifiers can be used for this purpose. In this work, we tested the use of linear classifiers [18], quadratic classifiers [19] and neural networks [20]. After several tests, we observed that the best results for this specific study were obtained using neural networks, specifically a multilayer perceptron (MLP) with 10 neurons, which represents a good tradeoff between computational cost after training and performance in terms of classification error rate. Figure 8 shows an example of the use of an MLP. An input vector x=[x(1),⋯,x(N)] composed of a set of *N* extracted features is used as the input. In this work, we use one hidden layer composed of 10 tan-sigmoidal neurons, and the number of components *M* of the output vector y=[y(1),⋯,y(M)] depends on the experiment and matches the number of classes in the problem at hand. The decision is a function of which term y(m) is maximal. The MLPs were trained using the Levenberg–Marquardt algorithm and using a validation set that provides an early stop in the training process to minimize the loss of generalization due to overtraining.

The proposed MLP classifier and the feature extraction system were implemented in the smartphone described in Section 2 using the full set of features, to assess whether the system is suitable for real-time applications using currently available smartphones. After four hours of full performance, the battery level decreased by an additional 10%, compared to the standard decrease of the battery level in sleep mode, using less than 6% of the CPU resources of the smartphone. Further experiments demonstrated that the battery consumption was primarily due to the Bluetooth communication with the ECGZ2 device, because similar results were obtained in the case of switching off the feature extraction and the classification blocks.

### 3.3. Feature Selection

Selection of a suitable reduced set of features is one of the key points in the design of classification systems for real-time stress analysis. The use of a large set of features (even the full set composed of 56 ECG-based features and 56 TEB-based features, totaling 112 features) might cause problems in the generalization ability of the obtained results, resulting in degraded system performance in real scenarios. For this purpose, we reduced the amount of features used in the classification based on the objectives: first, improving the performance to avoid overtraining and, second, reducing the computational complexity of the final solution.

Numerous algorithms are available that are aimed at selecting the best features from a global set, and in recent years, evolutionary algorithms have became a standard solution for solving this type of problem. These algorithms apply evolutionary laws to a generic population of possible solutions to meta-heuristically find the best possible solution [21,22]. In particular, genetic algorithms can be successfully applied to the problem of feature selection [23], and they have been successfully used in different research areas, such as speech processing [24] or optimization of an economic dispatch problem [25]. With these algorithms, it is possible to find the best features that achieve the maximum efficiency, which in our work is defined by low classification error with a constrained global number of operations per second $N_{op}$.

In our problem, we seek the best reduced set of features that aids the system in performing proper classification. For this purpose, a “population” of possible sets of features is evaluated with the goal of maximizing the percentage of correct classification. Because several possible solutions must be tested, we use linear classifiers that allow training and testing to be performed much faster than with NNs. Moreover, constraints can be easily added to genetic algorithms. In this case, we limited the maximum number of operations per second ($N_{op}<N_{max}$). Therefore, the algorithm must find the best set of features using less than $N_{max}$ operations per second.

To avoid loss of generalization in the results while maximizing accuracy in the estimation of the error rate, *k*-fold cross-validation was used in the experiments, with *k* set equal to the number of subjects available in the design database. Thus, the data were divided into *k* folds or subsets containing data from each subject, and each time, the registers from one given subject are used as a test set, with the data from the remaining k-1 subjects used for the design tasks. Thus, the design and test process for the global system (feature selection, classifier design and error estimation using *k*-fold cross-validation) is performed following the described procedure:

- In each iteration of the *k*-fold cross-validation process, the recordings from the *i*-th subject are selected as a test set, and the recordings from the remaining k-1 subjects are used as a design set.
- The design test is used to determine the best subset of features by applying a genetic algorithm. In this process, the following steps are described:
  1. A “population” of 200 combinations of features is randomly generated.
  2. If there are two combinations with exactly the same set of features, one of them is modified by randomly replacing one of the features.
  3. For each combination in the population, if the associated number of instructions per second $N_{op}$ is greater than the maximum $N_{max}$, then a feature is randomly removed. This step is repeated as many times as necessary. If all of the features of a given combination are removed, then one feature is added at random until the condition $N_{op}<N_{max}$ is satisfied.
  4. Each combination is ranked using the mean squared error of a least squares linear classifier measured using a (k-1)-fold cross-validation process over the corresponding design set.
  5. The best 20 combinations of the population are selected as “survivals” and are used to generate a new population of 200 new solutions using the random crossover of two parents.
  6. Mutations are added to the population by changing a feature with a probability of 10%. It is important to highlight that the best individual of each population remains unaltered. The process iterates in Step 2 until 100 generations are evaluated.
- To avoid a local minimum in the optimization method, the feature selection process using the genetic algorithm is repeated five times with random initial populations. The final combination is selected as the best combination of all final populations. This solution renders exceptionally good results with a required number of operations per second $N_{op}$ that is less than the constraining value $N_{max}$.
- Once the best combination is finally selected, the obtained reduced set of features is used to train an MLP with 10 neurons. The design set is randomly subdivided into a training set containing the recordings of 90% of the individuals in the database, and the remaining data are used as a validation set to control the training process using an early stop constraint. To avoid local minima, the full training process is repeated five times, and the best network in terms of error over the design set is selected.

## 4. Description of the Experiments

To evaluate the performance of our system, a complete database was created in which stress and emotions were elicited through different methods. The experiments used to create the database were run with k=40 subjects. The subjects consisted mainly of students and climbers aged 20 to 49 and included 12 females and 28 males. The total duration of the complete experiment was approximately 90 min. The experiments were recorded in a laboratory and used to generate a database.

All of the experiments were performed under the conditions of respect for individual rights and ethical principles that govern biomedical research involving humans, and written informed consent was obtained from all participants. The experiments were approved by the Research Ethics Committee at the University of Alcala, and the protocol number assigned to this experiment is CEI: 2013/025/201130624.

A set of segments extracted from several validated movies was used to elicit emotion/emotional stress [26], and a set of games based on mental arithmetic were used to elicit mental load [27,28,29]. Finally, to elicit physical load, the participant climbed up and down stairs for five minutes.

**Figure 9:**
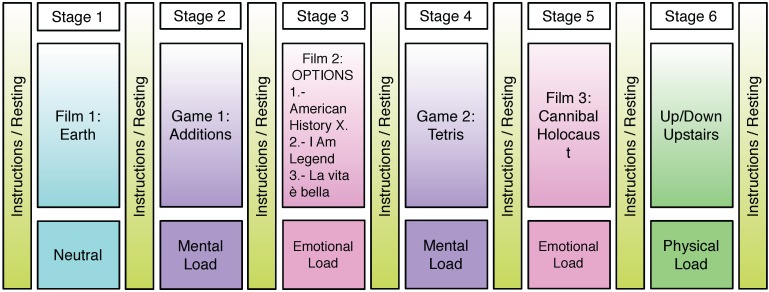
Scheme describing the stages of the experiment used to generate the database.

The experiment consists of six stages in which the subject must follow instructions given by a computer, with each stage consisting of a different task. Figure 9 shows the different stages, which are explained as follows:

- Stage 1: In this stage, the subject must watch a fragment of the nature BBC documentary film “Earth” two times. The fragment lasts from the timestamp 00:00:45 to 00:07:06. The first time, the subject simply watches the movie, and the second time, he/she must continuously note the felt emotion from a set of emotions, with the aim of assessing the elicited emotion. We labeled this stage as the neutral state.
- Stage 2: A game based on addition was specifically developed for the second stage. A sum of two digits is shown on the screen; the subject must do the sum mentally and click the correct solution from a set of possible solutions. The number of digits in the to sum increases from two to five every 25 sums. The difficulty of the task is automatically adapted to the ability of the user, such that if the subject responds correctly, less time is available for the next sum, and if he/she does not complete the sum in the time estimated, the time available for the next sum is increased. Each time that the user fails, a disgusting and stressful sound is played. The duration of this stage varies from five and seven minutes depending on the skill of the subject.
- Stage 3: In this stage, the subject has the option to choose among three different films, validated to elicit sadness:
  − “American History X” (1998) by Savoy Pictures, The Turman-Morriset Company (Christian, MO, USA) [30]. This movie is assumed to elicit strong emotional arousal in participants [31].
  − “I Am Legend” (2007) by Warner Bross. This movie is a post-apocalyptic science fiction/horror film [32]. The selected movie fragments were the most impactful such that they could induce a loaded emotional charge.
  − “Life is beautiful” (1997) by Miramax [33]. Again, the fragments used were representative in order to induce sadness [34].

In all cases, scenes with a total duration of approximately 12 min were selected, and each participant watched the selected movie twice. The first time, the subject simply watches the movie, and the second time, he/she must continuously note the felt emotion from a set of emotions, with the aim of assessing the elicited emotion.

- Stage 4: In this stage, the subject must play the well-known game “Tetris”. This game was used to elicit a workload in several papers, such as [35]. To intensify the mental load, we added stressful sounds and increased the rhythm of the game. The difficulty of the game varies from easiest to hardest and attempts to apply the highest work load to the subject. Thus, when the participant removes rows, the speed is slightly increased, and when he/she loses, the game restarts again with a speed slightly slower than the speed at the end of the previous game. The duration of this stage varies from 5 to 7 min depending on the skill of the subject.
- Stage 5: The last film fragment was selected from “Cannibal Holocaust” (1980) by F.D Cinematografica [36] and was intended to generate disgust, which represents a strong emotional load [37]. The duration of the fragments is close to three minutes, and again, each participant watches the selected movie twice.
- Stage 6: The aim of this stage is to check the functionality of the system (vest + algorithms) with the moving subject and to elicit physical workload. The experiment was performed at university facilities in a location with three floors. For this test, the subject must climb up and down the stairs quickly for 5 min.

As we can see in Figure 9, before each stage of the experiment, there is a baseline stage, denominated “instructions/resting”, which was primarily used to rest and to read the instructions of the next stage. This way, the subjects are not continuously experiencing mental, emotional or physical load, which could lead to a decrease in the performance. The length of this inter-stage interval was about 5 min approximately, the time required to achieve a stable hemodynamic condition [38]. Longer resting times have not been considered, since they would suppose an excessive total length of the experiment, which might fatigue the subjects.

To study the ability of the sensor-based system to analyze stress load, three different analyses of the generated database were considered:

- Analysis 1: In the first analysis, classification of the type of activity was used as the target of the analysis. For this purpose, four different situations were used as classes (M=4):
  − Neutral activity, which was registered during the last 140 s of the first movie, *i.e*., the documentary; thus, considering that each individual watched each movie twice, we obtained a total of 280 s for each individual in the database.
  − Emotional activity, which was characterized during viewing of the last 70 s of the second and third movies; therefore, we again obtained a total of 280 s per individual.
  − Mental activity, which was registered during the last 140 s of both games, produced 280 s in total.
  − Physical activity was registered during the last 280 s of the physical activity stage.
- Analysis 2: In the second analysis, the objective was to distinguish among the emotional states during the viewing of the movies, according to three different emotions (M=3): neutral emotion, sadness and disgust. For this purpose, recordings from the last 200 s of each movie were used, generating 400 s for the neutral emotion (the documentary), 400 s for sadness (the second movie) and 400 s for disgust (the third movie).
- Analysis 3: In the third analysis, the objective was to distinguish between low mental activity at the beginning of the games and high mental load at the end of the games (M=2). The first 110 s of each game were labeled as low mental activity, and the last 110 s of each game were labeled as high mental activity (mainly due to the different difficulty of the games from the beginning to the end).

Therefore, the database is fully balanced in all three analyses.

To assess elicitation of the SNS activity in the different analyses, Table 2 shows the average values for the IF signals in the different classes defined in the three analyses: type of activity, emotional state and mental activity. The two-sample Kolmogorov–Smirnov test, one of the most useful and general nonparametric methods for comparing two samples, was performed for each combination of two classes and for each IF signal. Statistically-significant differences (p<0.01, significance level of 0.05) were found in all comparisons performed for the $E_{\mathrm{RT}}$, $E_{\mathrm{RD}}$, $Z_{\mathrm{CF}}$ and $Z_{\mathrm{RT}}$ signals in the case of Analysis 1 and the $E_{\mathrm{RD}}$, $Z_{\mathrm{CF}}$ and $Z_{\mathrm{RT}}$ signals in the case of Analysis 2. These results demonstrate the correct elicitation of the emotions in the database.

**Table 2:** Average values for the eight IF signals in the different classes defined in the three analyses: type of activity, emotional state and mental activity.

IF Signal	Analysis 1				Analysis 2			Analysis 3	
	Type of Activity				Emotional State			Mental Activity	
	Neutral	Emotional	Mental	Physical	Neutral	Sad	Disgust	Low	High
$E_{\mathrm{CF}}$	4.92	4.93	4.92	5.36	4.92	4.93	4.92	4.92	4.92
$E_{\mathrm{RT}}$	28.17	29.62	27.69	24.75	27.95	29.53	28.46	28.76	27.59
$E_{\mathrm{RD}}$	0.32	0.40	0.45	1.20	0.32	0.43	0.43	0.44	0.45
$E_{\mathrm{PPM}}$	72.07	70.99	74.62	130.43	72.10	70.21	71.12	74.37	74.77
$Z_{\mathrm{CF}}$	5.61	6.33	5.74	7.89	5.60	5.74	6.89	5.75	5.72
$Z_{\mathrm{RT}}$	19.44	20.10	20.50	27.17	19.45	19.85	20.27	20.97	20.46
$Z_{\mathrm{RD}}$	0.09	0.16	0.27	0.67	0.09	0.09	0.23	0.19	0.25
$Z_{\mathrm{PPM}}$	74.36	71.99	74.30	93.86	74.03	72.62	71.80	74.34	74.35

## 5. Results

This section discusses the results obtained by the proposed system in the different analyses. In the first approach, the results are shown as a function of classification error probability *versus* number of operations per second ($N_{op}$), for different values of $N_{max}$ (maximum number of operations per second).

### 5.1. Analysis 1: Activity Identification

**Figure 10:**
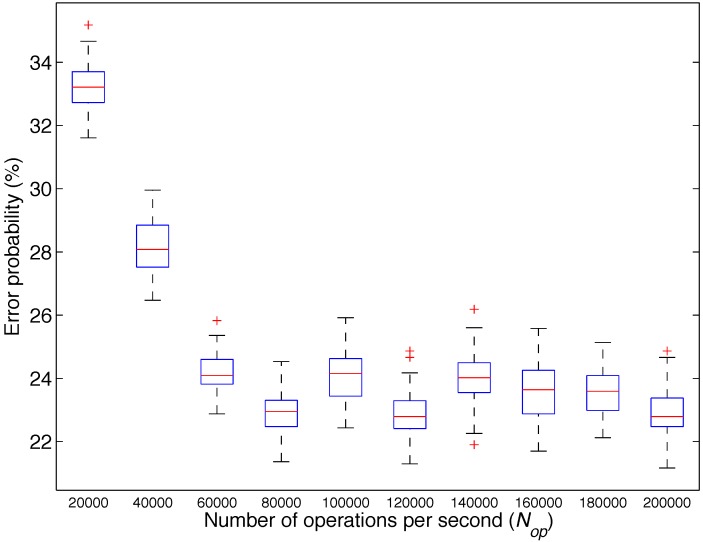
Box plot of the probability of error obtained by the classifiers for different values of the maximum number of operations per second ($N_{op}$) particularized for Analysis 1: type of activity.

In the first analysis, we used four different types of activities: neutral, emotional, mental and physical. The algorithm described in Section 3.3 was applied 100 times, for values of $N_{max}$ from 20,000 to 200,000 in steps of 20,000. Figure 10 shows the box plot of the obtained results, which represent the classification error probability *versus* the number of operations per second $N_{op}$, for different values of $N_{max}$. We observe that for 20,000 $N_{op}$, the mean error probability obtained is approximately 33%. The error probability decreases as the value of $N_{op}$ increases up to 80,000.

To illustrate the obtained results and the differences of classification errors between classes in detail, Table 3 shows the confusion matrix and percentage of classification error (error probability) for each class defined in Analysis 1 using a maximum number of 80,000 operations per second ($N_{op}<80,000$). The confusion matrix shows how the classifier encounters additional difficulty in distinguishing between emotional and mental classes, with physical activity as the most easily recognizable class.

The average error obtained with 80,000 $N_{op}$ is approximately 21.23%, corresponding to the use of approximately 12 features. All features selected by the genetic algorithm are based on the ECG measurement. More concretely, the main features used are the mean, the standard deviation, the kurtosis, the minimum, the skewness and the baseline of the $E_{\mathrm{CF}}$ signal, and the mean, the standard deviation, the harmonic mean and the baseline of the $E_{\mathrm{PPM}}$ signal.

**Table 3:** Confusion matrix and percentage of classification error (error probability) for each class using a maximum number of 80,000 operations per second ($N_{op}<80,000$), particularized for Analysis 1: type of activity.

True Class	Predicted Class				% Error Probability
	Neutral	Emotional	Mental	Physical	
Neutral	971	3	146	0	13.30%
Emotional	12	881	199	28	21.34%
Mental	128	374	604	14	46.07%
Physical	0	37	10	1073	4.20%
Average					21.23%

For this first analysis, the obtained average error rate in this real-time activity identification is 21.23%, which is slightly better than those obtained from the heart rate (23.72%) and the respiration rate (24.40%) in [9] and significantly better than the 31% error rate obtained from HRV analysis in [39].

### 5.2. Analysis 2: Emotional State

**Figure 11:**
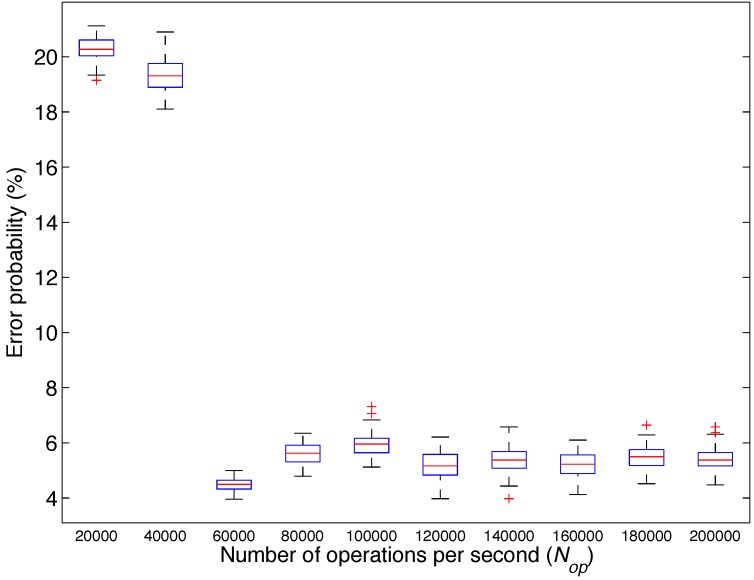
Box plot of the probability of error obtained by the classifiers for different values of the maximum number of operations per second ($N_{op}$) particularized for Analysis 2: emotional state.

In the second analysis, the objective was to distinguish among three emotions: neutral emotion, sadness and disgust. Again, the algorithm described in Section 3.3 was performed 100 times for values of $N_{max}$ from 20,000 to 200,000 in steps of 20,000. Figure 11 shows the box plot of the obtained results and represents the classification error probability for different values of $N_{max}$. We observe that the mean error probability obtained is the lowest for 60,000 $N_{op}$.

Table 4 shows the confusion matrix and percentage of classification error (error probability) for each class of the second analysis using a maximum of 60,000 operations per second ($N_{op}<60,000$). In this case, the average error probability obtained is 4.77%, and the confusion between sadness and disgust is highlighted. The main features selected by the genetic algorithm in this case are the mean and the baseline of the $E_{\mathrm{CF}}$ signal and the mean and the baseline of the $Z_{\mathrm{PPM}}$ signal.

**Table 4:** Confusion matrix and percentage of classification error (error probability) for each class using a maximum number of 60,000 operations per second ($N_{op}<60,000$), particularized for Analysis 2: emotional state.

True Class	Predicted Class			% Error Probability
	Neutral	Sadness	Disgust	
Neutral	1584	16	0	1.00%
Sadness	12	1496	92	6.50%
Disgust	0	109	1491	6.81%
Average				4.77%

For this analysis, the obtained error rate is 4.77%, which is a result considerably lower than the 14% error rate obtained in [40], in which facial electromyogram, respiration, electrodermal activity and electrocardiogram signals are used to classify five emotional classes (high stress, low stress, disappointment, euphoria and neutral face).

### 5.3. Analysis 3: Mental Activity

**Figure 12:**
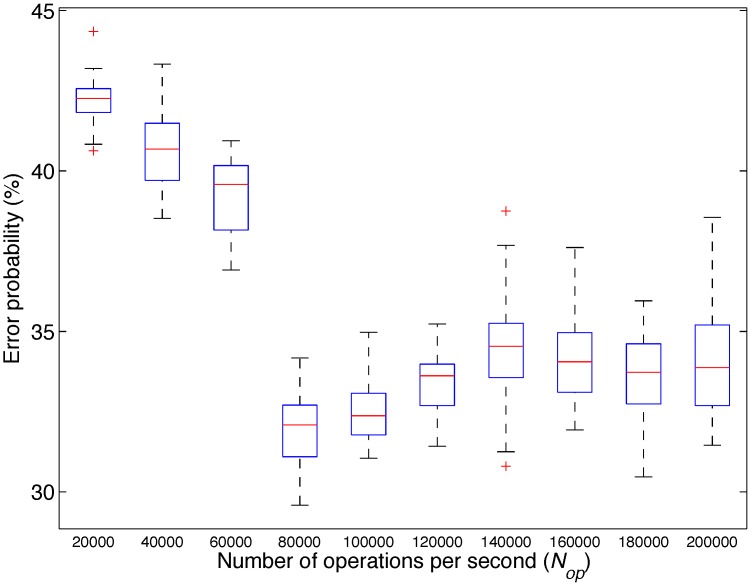
Box plot of the probability of error obtained by the classifiers for different values of the maximum number of operations per second ($N_{op}$) particularized for Analysis 3: mental activity.

This analysis contains two different classes: low and high mental activity. Figure 12 displays the obtained results. Once again, low $N_{op}$ produces the highest error and an increase of $N_{op}$ reduces the error. In this case, the best performance is obtained for 80,000 $N_{op}$.

Table 5 shows the confusion matrix and percentage of classification error (error probability) for each class using a maximum number of 80,000 operations per second ($N_{op}<80,000$), specific to Analysis 3: mental activity. In this case, the average error probability obtained is 32.33%. The main features selected by the genetic algorithm in this case are the minimum and the maximum of the signal $Z_{\mathrm{RT}}$; the mean, the skewness, the harmonic mean and the baseline of $Z_{\mathrm{CF}}$; the minimum, the maximum, the skewness, the kurtosis and mean absolute deviation of $Z_{\mathrm{RD}}$; and the baseline of $E_{\mathrm{CF}}$.

**Table 5:** Confusion matrix and percentage of classification error (error probability) for each class using a maximum number of 80,000 operations per second ($N_{op}<80,000$), particularized for Analysis 3: mental activity.

True Class	Predicted Class		% Error Probability
	Low Mental Load	High Mental Load	
Low mental load	572	308	35.00%
High mental load	261	619	29.66%
Average			32.33%

In this third analysis, the obtained average error rate is 32.33%. The error rate for a similar analysis was higher using ECG or EOG (Electrooculogram) signals [41], but significantly lower (14% error rate) when the EEG signal was used.

## 6. Conclusions

Analysis of the ECG and TEB measurements recorded with sensorized instrumentation can be highly useful for detecting high levels over long periods of time or sudden increases in mental overload, emotional response or physical activity for workers in professions with associated risks, such as fire-fighters, first responders, police and soldiers, among others. The designed software performs quite well with $N_{op}=60,000$ operations per second for the emotional state experiments (approximately 5% of estimated classification error) and with $N_{op}=80,000$ operations per second for the determination of the type of activity (approximately 21% of estimated classification error) and mental overload (approximately 32% of estimated classification error). In two out of the three classification task, the feature selection algorithm selected features obtained from both ECG and TEB physiological measurement. For task identification, only information from the ECG measurement was selected. This real-time solution was successfully implemented in an actual smartphone.

The current wearable biomedical measurements and smartphone technologies allow realistic implementation of personalized monitoring systems for the detection of stress, mental overload and emotional status in real time. The combination of these currently existing technologies could potentially enable solutions for monitoring of signs related to the development of stress-related diseases at work or prompt detection of acute increases in stress levels in specifically dangerous job scenarios. Such specialization would require further investigations to trim the classification engine to target the new required specifications. Although, these tasks are certainly not trivial, from the current stand point, they appear completely feasible.
